# Meteorological extremes and their impact on tinnitus-related emergency room visits: a time-series analysis

**DOI:** 10.1007/s00405-023-07894-1

**Published:** 2023-03-01

**Authors:** Markus Haas, Mateo Lucic, Franziska Pichler, Alexander Lein, Faris F. Brkic, Dominik Riss, David T. Liu

**Affiliations:** Department of Otorhinolaryngology, Head and Neck Surgery, Medical University of Vienna, Waehringer Guertel 18–20, 1090 Vienna, Austria

**Keywords:** Emergency service, hospital, Epidemiology, Tinnitus, Ear, Weather

## Abstract

**Purpose:**

Extreme weather events are rising due to the accelerating pace of climate change. These events impact human health and increase emergency room visits (EV) for many morbidities. Tinnitus is a common cause of EVs within otolaryngology in Germany and Austria. The effect of extreme weather conditions on tinnitus-related EVs is unknown.

**Methods:**

A total of 526 tinnitus-related EVs at a tertiary care hospital in Vienna were identified. A distributed lag non-linear model with a maximum lag period of 14 days was fitted to investigate the immediate and delayed effect of single-day and prolonged (three-day) extreme atmospheric pressure, relative humidity, mean temperature, precipitation and mean wind speed on EV rates. Extreme conditions were defined as the 1st, 5th, 95th, and 99th percentile of the meteorological variables. Relative risk (RR) is defined as risk for tinnitus-related EVs at an extreme condition compared to the risk at the median weather condition. Cumulative RR (cRR) is the total cumulated EV risk for a given time period.

**Results:**

High relative humidity increased same-day RR for tinnitus-related EVs to 1.75. Both low and high atmospheric pressure raised cRR as early as three days after an event to a maximum of 3.24. Low temperatures mitigated cRR within 4 days, while high temperatures tended to increase risk. Prolonged precipitation reduced cRR within one day.

**Conclusion:**

Extreme meteorological conditions are associated with tinnitus-related EV rates. Further investigation into potential causative links and underlying pathophysiological mechanisms is warranted.

**Supplementary Information:**

The online version contains supplementary material available at 10.1007/s00405-023-07894-1.

## Introduction

The latest Intergovernmental Panel on Climate Change report paints a devastating picture of the current trajectory of the global climate, describing a sharp rise in extreme weather events [1]. Heat waves and increased climate variability negatively affect human health and lead to higher mortality and morbidity [2]. Emergency departments constitute the first point of contact in managing diseases affected or exacerbated by extreme weather events, resulting in a considerable increase in emergency room visits (EV) during and after such conditions [3].

Tinnitus is the perception of sound without an external stimulus. Although frequently idiopathic, it may also be the first manifestation of severe health issues [4, 5]. Recent studies estimate that, worldwide, 740 million adults are affected by tinnitus, with an annual incidence of approximately 1%, making its global burden comparable to that of pain and migraine [6]. About one in 50 patients suffering from tinnitus experience severe symptoms that require urgent medical attention [6]. From an otorhinolaryngological perspective, tinnitus is a common reason for emergency department visits and ranked third at 4.9% of all ear, nose and throat (ENT)-related EVs a in recent analysis of ENT emergencies in Germany [7].

Hearing loss, chronic noise exposure, stress and psychiatric comorbidities, particularly depression and anxiety, are regarded as important risk factors for tinnitus [5, 8, 9]. Patients with normal hearing and tinnitus may still display subclinical anomalies during audiological testing [10]. The pathophysiology of tinnitus is complex and involves several levels of the auditory pathway. According to the current European tinnitus guidelines, potential peripheral mechanisms include activation of cochlear *N*-methyl-d-aspartate receptors, outer hair cell alterations and shifts in endo-cochlear potentials [11–13]. Proposed central mechanisms range from central hyperactivity following hearing loss through neural homeostatic plasticity to cortical tonotopic remapping and hyperpolarization of thalamic neurons due to reduced sensory inputs [13, 14]. Recent studies even reported an association between tinnitus and COVID-19 [15, 16]. Given the plethora of potential underlying mechanisms and risk factors, tinnitus remains difficult to treat. Cognitive behavioral therapy is currently the only recommended treatment by European and American guidelines [13, 17].

EV rates for psychiatric (anxiety, mood disorders) and somatic risk factors (hypertension, diabetes) for tinnitus are associated with extreme weather events [18–20]. However, the current literature on weather-associated tinnitus occurrence is limited to Ménière's disease (MD), a distinct inner ear disorder characterized by a symptom triad of tinnitus, hearing loss and vertigo. Low atmospheric pressure and high humidity have been reported to increase the risk of a Ménière’s attack. In contrast, atmospheric pressure and mean temperature have been correlated with tinnitus severity [21]. However, the impact of extreme weather events on tinnitus as a primary symptom, independent of MD, remains to be investigated. Furthermore, we currently lack an understanding of how delayed effects of extreme meteorological events impact tinnitus beyond the same day. Identifying environmental factors to predict tinnitus-related EV rates has potential implications for improving the resilience of healthcare systems towards a changing climate and optimizing health resource management. In addition, the possible correlation with weather conditions could provide further insight into tinnitus pathophysiology.

In the current study, we analyzed tinnitus-related EVs at a tertiary care hospital in Austria employing a distributed lag non-linear model (DLNM) to investigate the immediate and delayed effects of extreme weather events.

## Materials and methods

### Study population and meteorological data

A total of 526 tinnitus-related EVs were identified by screening the electronic patient records of the Vienna General Hospital’s emergency room (ENT division) from January 1st, 2015, to December 31st, 2018, for EVs mentioning tinnitus as a current symptom. Only patients with tinnitus as a primary symptom for their EV, absence of suspicion for or former diagnosis of MD, lack of signs for ear infections or labyrinthitis and lack of reported acute hearing loss as a primary symptom were included. Basic patient information (date of visit, age, sex, referral status, admission status) and relevant clinical information regarding diagnostic workup (laterality, results of Weber’s and Rinne’s test, suspected etiology and blood pressure at presentation) were extracted for every tinnitus-related EV.

Meteorological data for Vienna was provided by the national meteorological and geophysical service of Austria (Central Institution for Meteorology and Geodynamics—“Zentralanstalt für Meteorologie und Geodynamik”) from January 1st, 2015, to December 31st, 2018, including the daily atmospheric pressure, relative humidity, mean temperature, precipitation and mean wind speed. All measurements were taken within three kilometers of the Vienna General Hospital (elevation: 177 m above sea level, latitude: 48.198°; longitude: 16.3669°).

### Statistical analysis

The immediate and delayed effects of extreme weather conditions on tinnitus-related EV rates were analyzed using a DLNM [22], an established model commonly used to quantify the effects of meteorological conditions and air pollution on mortality and morbidity [23, 24]. The meteorological conditions were used as independent variables. The total number of daily tinnitus-related EVs was chosen as the response variable. We describe the relationship between meteorological conditions and lags (lag days, i.e., the days following the first exposure to a certain weather condition) using natural cubic splines with five degrees of freedom (df) at equally spaced quantiles for daily meteorological conditions and equal intervals on the logarithmic scale of lags [25]. To account for potential harvesting effects, we chose a maximum lag period of 14 days [26]. Hereby, we control for a potential decrease in EV rates after extreme weather events trigger tinnitus episodes in vulnerable patients, who are less likely to seek emergency care soon after an initial visit, where they are referred to follow-up diagnostics and treatment. Natural cubic splines of time with seven *df* per year were employed to control for seasonality and long-term trends. We included a weekday indicator variable in the model to account for differing demand throughout the week (e.g., due to the closing of doctor’s offices, and external providers on weekends). We used a dummy variable to control for public holidays due to the expected increase in demand during those days. Akin to weekends, general practitioner’s offices, ENT doctor’s offices and outpatient ENT departments of hospitals are closed during Austrian public holidays. Thus, emergency rooms remain the sole providers during those days, which is expected to increase visitation rates. As public holidays and weekend days sometimes coincide, which may reduce the effect of public holidays on EV rates, an interaction between the weekday indicator and the public holiday dummy was included. According to the coefficient estimates, EVs were constant during the week, with a statistically significant increase in EVs on public holidays, Fridays, Saturdays and Sundays. The interaction coefficients between weekend days and public holidays were negative for Saturdays and Sundays and statistically significant for Saturdays. Hence, tinnitus-related EVs are not expected to increase further when public holidays and Saturdays coincide. Furthermore, we controlled for a procedural change in emergency room admissions using a dummy variable after the introduction of a pre-screening outpatient department in December 2016. The estimated coefficient did not show statistical significance. Finally, we calculated the Pearson correlation coefficient for every combination of the five independent weather variables. Temperature and relative humidity showed a strong negative correlation, while all other combinations showed weak to no correlation.

Extreme weather events were defined as the 1st, 5th, 95th, and 99th percentile of atmospheric pressure, relative humidity, mean temperature and mean wind speed and the 95th and 99th percentile for precipitation. The median of each weather condition was used as a reference for calculating relative risk (RR) for each lag day. RR describes the risk for an increase or decrease in total daily emergency room cases with tinnitus as their chief complaint on a given lag day at extreme conditions compared to median conditions. The cumulative relative risk (cRR) was calculated by the cumulation of RR from lag0 up to lag14. cRR describes the cumulated risk for tinnitus-related EV after extreme weather events compared to the EV risk at the median weather condition within a stated period.

Next to single-day events, we investigated risk after prolonged three-day long extreme weather events. The effects of prolonged events were calculated using a rolling window. The number of tinnitus-related EVs over the previous three days acted as the response variable. Lag0 refers to the last day of the three-day window. The 1st, 5th, 95th, and 99th percentile of the three-day mean for atmospheric pressure, relative humidity, mean temperature and mean wind speed and the 95th and 99th percentile of the sum of precipitation over three days were defined as prolonged extreme conditions.

Numeric values for RR (single-day: Supplementary Table 1; prolonged: Supplementary Table 3) and cRR (single-day: Supplementary Table 2; prolonged: Supplementary Table 4) were extracted at day 0, day 1, day 3, day 7 and day 14. RR and cRR values are reported with their 95% confidence interval (CI) and p-value throughout the manuscript.

R software (version 4.1.3) was used to perform statistical testing [27]. The DLNM was fitted using the R package “dlnm” [28].

## Results

### Study population and weather

**Fig. 1:**
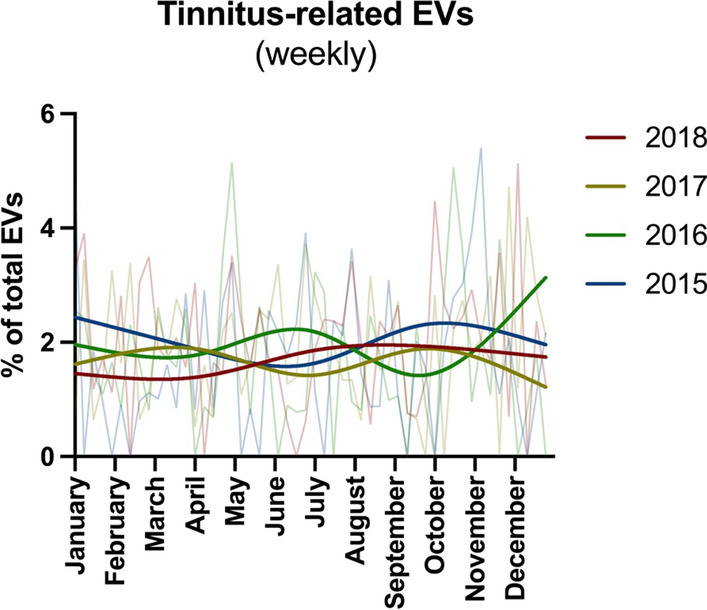
Weekly tinnitus-related EVs are shown as the percentage of total EVs from any cause from 2015 to 2018 using smoothing splines (lines) and daily values (background)

From January 1st, 2015, to December 31st, 2018, 526 tinnitus-related EVs occurred at the Vienna General Hospital in Vienna, Austria. Patient characteristics for each EV are shown in Table 1. The study population had a higher proportion of male patients (58.4%) with a median age of 37 (range: 11–89). Most patients suffered from one-sided tinnitus (75.5%) and showed no indication of hearing loss, as determined by Weber’s and Rinne’s test (80.4%). In those with pathologic results, 15.7%, 1.3%, and 2.6% of patients showed signs of sensorineural loss, conductive loss and combined loss, respectively. The etiology was largely idiopathic (95%). However, some cases were attributable to recent acoustic trauma (2.5%) and cerumen (2.5%). From the small number of cases with available blood pressure measurements (*n* = 88), 55.7% of patients were hypertensive. On average, 2.5 tinnitus-related EVs occurred per week. Daily presentation rates throughout 2015–2018 are shown in Fig. 1. While the highest numbers of tinnitus-related EVs occurred during January (*n* = 55) and October (*n* = 54), no clear seasonal trends were observed. All meteorological variables in Vienna during the observational period are shown in Supplementary Fig. 1. Relative humidity and mean temperature showed clear differences between the seasons. Atmospheric pressure, precipitation and mean wind speed were less affected by seasonality.

**Table 1:** Patient characteristics of the study cohort

Patient characteristics for tinnitus-related EV		
	*n* (% missing)	Count (%)
**Age**	526 (0%)	–
0–18	–	19 (3.6%)
19–29	–	165 (31.4%)
30–44	–	155 (29.5%)
45–64	–	141 (26.8%)
65+	–	46 (8.7%)
**Sex**	526 (0%)	–
Male	–	307 (58.4%)
Female	–	219 (41.6%)
**Referral by an external provider**	526 (0%)	–
Yes	–	83 (15.8%)
No	–	443 (84.2%)
**Admission required**	526 (0%)	–
Yes	–	3 (0.1%)
No	–	523 (99.9%)
**Laterality of tinnitus**	458 (12.9%)	–
One-sided	–	346 (75.5%)
Two-sided	–	112 (24.5%)
**Weber’s and Rinne’s test**	312 (40.7%)	–
No indication of hearing loss	–	251 (80.4%)
Indicating sensorineural loss	–	49 (15.7%)
Indicating conductive loss	–	4 (1.3%)
Indicating combined loss	–	8 (2.6%)
**Etiology**	526 (0%)	–
Idiopathic	–	496 (95.0%)
Acoustic trauma	–	13 (2.5%)
Cerumen	–	13 (2.5%)
**Arterial hypertension at presentation**^a^	88 (83.3%)	–
Yes	–	49 (55.7%)
No	–	39 (44.3%)

^a^Systolic pressure > 140 mmHg

### Atmospheric pressure

Based on previous reports on the link between atmospheric pressure and tinnitus symptoms in patients suffering from MD [21, 29], we first aimed to investigate the effect of extreme atmospheric pressure events on tinnitus symptoms in patients without MD.

**Fig. 2:**
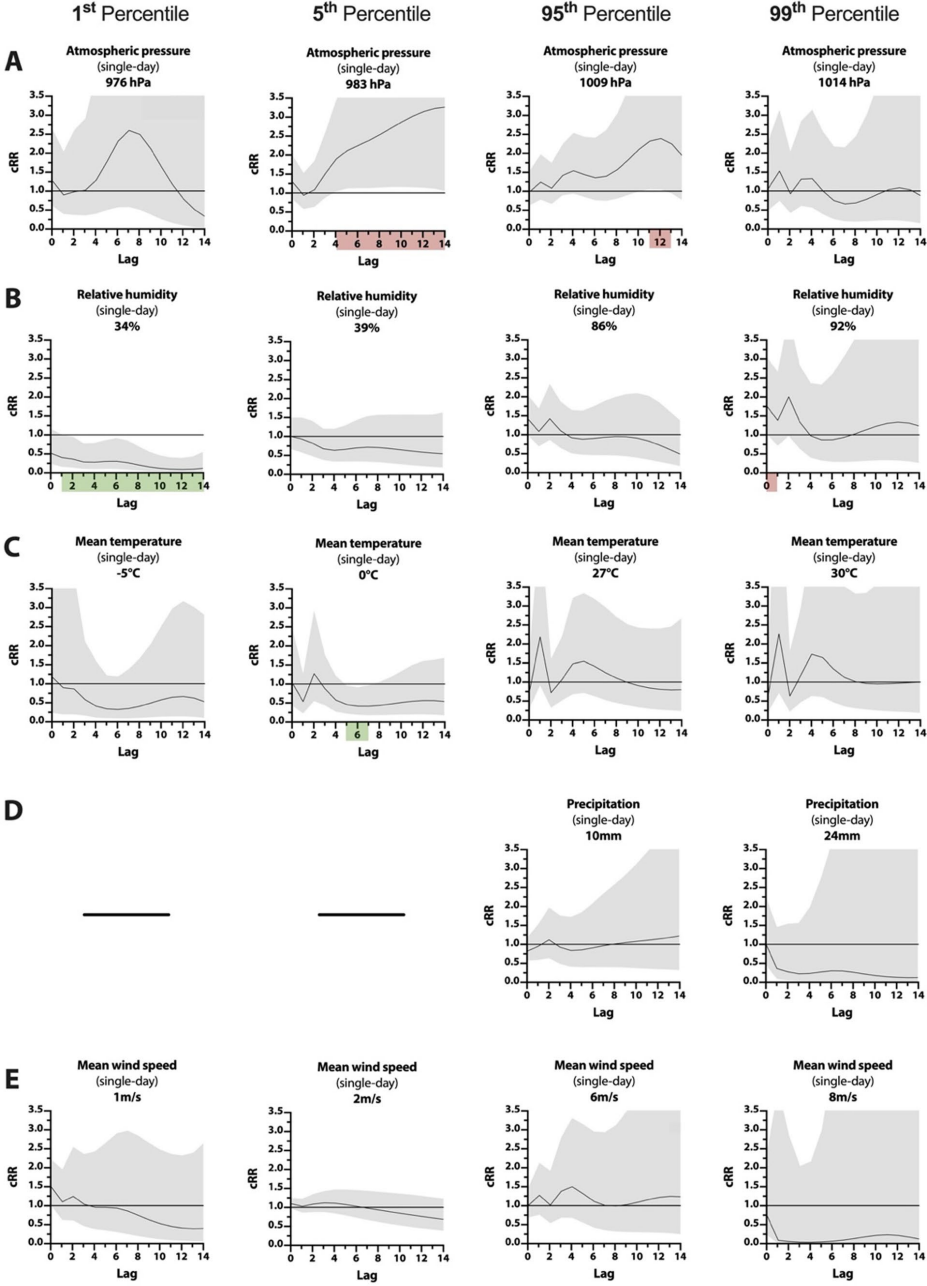
Line-plots of cRR for tinnitus-related EV are shown for single-day extreme weather events from lag0 to lag14 defined as the 1th, 5th, 95th, and 99th percentile of atmospheric pressure in hPa (**A**), relative humidity in % (**B**), mean temperature in °C (**C**), precipitation in mm [P_95_ & P_99_ only] (**D**) and mean wind speed in m/s (**E**). Confidence intervals (95%) are shown in grey. Significant decreases (green) and increases (red) in cRR (*p* ≤ 0.05) are highlighted on the lag-axis

On the same day, extreme atmospheric pressure events did not significantly affect tinnitus-related EV. Over the subsequent lag period of single-day events, the earliest impact of extremely low atmospheric pressure occurred after 3 days at 983 hPa (P_5_) with an RR of 1.40 [1.04–1.89; *p* = 0.028]. For the same condition, cRR was elevated within 4 days to 1.88 [1.03–3.44; *p* = 0.038] and was significantly elevated for the remaining lag period with the highest cRR of 3.24 [1.04–10.15; *p* = 0.044] at day 14 (Fig. 2A). Similarly, extremely high atmospheric pressure at 1009 hPa (P_95_) increased RR to 1.12 [1.01–1.25; *p* = 0.028] starting at day 8 and elevated cRR to 2.31 [1.04–5.10; *p* = 0.038] on day 11.

**Fig. 3:**
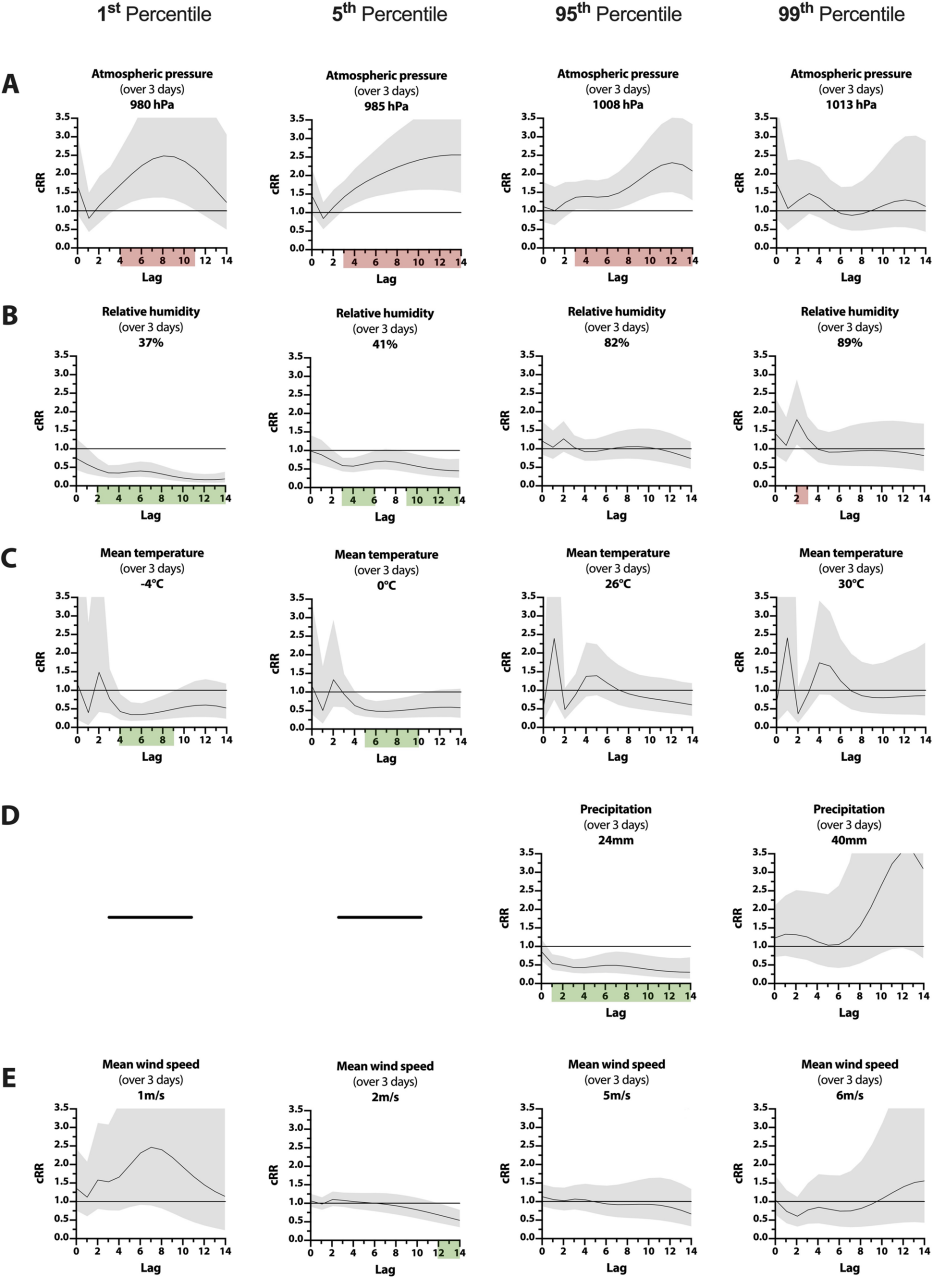
Line-plots of cRR for tinnitus-related EV are shown for prolonged extreme weather events over three days from lag0 to lag14 defined as the 1th, 5th, 95th, and 99th percentile of atmospheric pressure in hPa (**A**), relative humidity in % (**B**), mean temperature in °C (**C**), precipitation in mm [P_95_ & P_99_ only] (**D**) and mean wind speed in m/s (**E**). Confidence intervals (95%) are shown in grey. Significant decreases (green) and increases (red) in cRR (*p* ≤ 0.05) are highlighted on the lag-axis

Prolonged extremely low atmospheric pressure showed comparable results with the earliest elevation of cRR at 980 hPa (P_1_) to 1.7 [1.09–2.66; *p* = 0.020] within 4 days and at 985 hPa (P_5_) to 1.42 [1.09–1.85; *p* = 0.010] within 3 days, respectively (Fig. 3A). Following the single-day results, extremely high atmospheric pressure over 3 days at an average 1008 hPa (P_95_) raised the cRR to 1.36 [1.04–1.77; *p* = 0.026] within 3 days and led to a sustained cRR elevation over the remaining 14-day observational period with its maximum on day 13 at 2.23 [1.43–3.47; *p* < 0.001].

Taken together, atmospheric pressure increased tinnitus-related EV risk at low and high extremes as early as 3 days after the weather event.

### Relative humidity

After we found extreme atmospheric pressure conditions to be a risk-increasing factor for tinnitus-related EV, we then analyzed the impact of relative humidity due to its correlation with Ménière’s attacks [21].

On the same day, extremely high relative humidity at 92% (P_99_) increased the risk for tinnitus-related EVs to 1.75 [1.01–3.03; *p* = 0.046]. Over the subsequent lag period for single-day events, extremely low relative humidity at 34% (P_1_) significantly decreased RR between 8 and 11 days after the event. cRR at 34% (P_1_) was reduced within 1 day to 0.40 [0.16–1.00; *p* = 0.050] and remained significantly decreased over the entire 14-day observational period with a cRR as low as 0.09 [0.02–0.39; *p* = 0.002] by day 12 (Fig. 2B). Extremely high relative humidity had no significant delayed effect.

Prolonged extremely low relative humidity over 3 days impacted the risk for tinnitus-related EV in a comparable way, with a significantly reduced RR between day 8 and day 12. cRR was decreased within 2 days to 0.44 [0.26–0.74, *p* = 0.002] at − 4 °C (P_1_) and within 3 days to 0.59 [0.43–0.79; *p* < 0.001] for 0 °C (P_5_). (Fig. 3B) An extremely high three-day average relative humidity of 89% (P_99_) decreased RR to 0.77 [0.62–0.95; *p* = 0.016] at day 4. However, cRR was significantly increased to 1.78 [1.11–2.85; *p* = 0.018] within 2 days after a prolonged extremely high humidity of 89% (P_99_).

In summary, extremely low relative humidity showed a pronounced reductive effect on tinnitus-related EV as early as one day after the event. Conversely, extremely high relative humidity significantly increased same-day risk and showed a bidirectional impact over the subsequent lag period.

### Mean temperature

Next, we investigated mean daily temperature as a possible factor in tinnitus-related EV rates based on its described role in tinnitus severity in MD [21].

On the same day, extreme temperature events did not significantly affect EV risk. Over the subsequent lag period of single-day events, extremely low temperatures of 0 °C (P_5_) led to a decreased RR of 0.66 [0.47–0.92; *p* = 0.014] on day 4 and a decreased cRR from day 5 to day 7 with a low-point of 0.41 [0.19–0.91; *p* = 0.028] at day 6 (Fig. 2C). Extremely high temperatures of 27 °C (P_95_) significantly affected RR in both directions across different lag days, with a reduction to 0.33 [0.12–0.93; *p* = 0.036] on day 2 and an increase to 1.39 [1.01–1.91; *p* = 0.044] on day 4. cRR was not significantly impacted by extremely high temperatures.

Prolonged extreme conditions over three days in the form of cold waves exerted a risk-mitigating effect on tinnitus-related EVs (Fig. 3C). The highest reduction of RR was observed on day 4 to 0.68 [0.46–1.00, *p* = 0.048] after cold waves averaging 0 °C (P_5_). cRR was reduced after cold waves averaging both − 4 °C (P_1_) and 0 °C (P_5_) within 4 to 10 days. The lowest risk was observed at − 4 °C (P_1_) within 6 days at a cRR of 0.34 [0.18–0.64, *p* < 0.001]. Heat waves affected RR for tinnitus-related EV in both directions, although the risk-increasing effect was more pronounced. At day 3, RR was increased to 1.83 [1.02–3.30; *p* = 0.044] for 26 °C (P_95_) and to 2.61 [1.15–5.89; *p* = 0.022] for 30 °C (P_99_). cRR was not significantly affected by heatwaves.

In brief, extremely low temperatures reduced the risk for tinnitus-related EV as early as 4 days after the weather event. Extremely high temperatures showed a non-linear effect on RR starting 2 days after an event with a tendency towards increased risk.

### Precipitation

Since the effect of precipitation on tinnitus has not yet been described, we chose to analyze it as an additional potential meteorological factor in tinnitus-related EV rates.

For single-day events, extremely high precipitation had no significant effect on same-day risk, nor did it show delayed effects in the subsequent 14-day observational period. (Fig. 2D) On the other hand, prolonged extremely high precipitation over 3 days had a risk-mitigating effect on tinnitus-related EV. At its earliest, RR was decreased to 0.62 [0.41–0.94; *p* = 0.026] 1 day after prolonged precipitation totaling 24 mm (P_95_). cRR for the same condition was decreased within 1 day to 0.53 [0.36–0.78, *p* = 0.002] and remained significantly reduced over the entire lag period (Fig. 3D). Late effects of prolonged precipitation totaling 40 mm (P_99_) moderately increased RR between day 8 and day 11 (*p* < 0.050). However, these effects did not remain significant after cumulation.

Therefore, the data indicates a risk-reducing effect of prolonged, but not single-day, extreme precipitation for tinnitus-related EV as early as 1 day after the weather event.

### Mean wind speed

Finally, we investigated mean wind speed, which does not appear to affect tinnitus in MD [21], to dissect potential differences in weather impact in tinnitus unrelated to MD.

On the same day, extreme wind speeds did not significantly impact risk for tinnitus-related EV. Over the subsequent lag period of single-day events, the earliest effect of extremely high wind speeds was observed at 6 m/s (P_95_) on day 3 with a RR of 1.36 [1.01–1.84, *p* = 0.046]. However, these effects did not remain significant after risk cumulation. (Fig. 2E) Extremely low wind speeds showed no significant impact on RR or cRR.

Prolonged extremely low wind speeds at 2 m/s led to a decreased RR between day 8 and day 14, with a low-point of 0.88 [0.81–0.95, *p* < 0.001] by day 14. cRR was significantly reduced by day 12 to 0.67 [0.46–0.98, *p* = 0.040] (Fig. 3E). Extremely high wind speeds over 3 days only showed late, marginal effects on RR after day 9, with no significant results after cumulation.

To summarize, extremely high single-day wind speeds had minor, early effects on tinnitus-related EV risk by day 2. In contrast, extremely low prolonged wind speeds led to a moderate, late reduction of EV risk starting at day 8.

## Discussion

In this study, we investigated the effect of extreme meteorological conditions on tinnitus-related EVs. Previous reports on meteorological effects on tinnitus are limited to study populations suffering from MD. Here, we report both parallels and differences in the impact of weather on EV risk for tinnitus without signs of MD.

Atmospheric pressure has been the best-described meteorological factor influencing MD attacks and tinnitus severity. Higher atmospheric pressure was positively correlated with the diagnosis of MD in a large-scale study analyzing over 7000 cases in South Korea [30]. Another prospective study identified increases in atmospheric pressure as risk factors for an MD episode on the following day [29]. More specifically for tinnitus, a study utilizing a mobile app for self-reporting symptoms for MD patients showed that low atmospheric pressure was associated with increased tinnitus severity [21]. In our study, extremely low and high atmospheric pressure led to delayed increases in tinnitus-related EV risk as early as two days after an event. The possible underlying mechanisms at work are likely multifactorial. For one, associated risk factors for tinnitus, such as headaches [31] and depressive symptoms [32], have been correlated with atmospheric pressure. They may, therefore, indirectly promote tinnitus symptoms. On the other hand, environmental air pressure may disturb the tympanic pressure equilibrium, affecting tinnitus cases with underlying middle and inner ear etiologies. An animal model investigating middle-ear and intracranial pressure and its effect on electrocochleographic response has shown that static middle-ear pressure and an unimpaired eustachian tube are vital in maintaining normal cochlear function [33]. A recent study demonstrated that pressure changes in the external auditory canal can result in middle ear muscle contractions and eustachian tube dysfunction, which, in turn, can lead to tinnitus [34]. With regards to direct cochlear effects, atmospheric pressure changes have been shown to cause alterations in the cochlea of rats including partial loss of stereocilia [35]. Loss of stereocilia may impair the function of outer hair cells (OHC) and lead to aberrant spontaneous activity of the cochlea, which is one of the current hypotheses for the pathophysiology of cochlear tinnitus [13]. Spontaneous activity and prolonged depolarization of inner hair cells is thought to arise from an excitatory shift following stereocilia damage or degeneration of OHC, detachment of OHC from the tectorial membrane or from pressure increases in the scala media [12, 36]. Although no conclusions regarding tinnitus pathophysiology can be drawn from our study, the reported associations between atmospheric pressure extremes and EV rates lend further support to the previously reported links between atmospheric pressure and tinnitus. Nevertheless, since the exact mechanisms still need to be fully understood, further investigation is needed to better explain the association between atmospheric pressure and tinnitus both in MD- and non-MD-related cases.

Another link has been drawn between high relative humidity and increased frequency of Ménière’s attacks and tinnitus [21, 30]. Our study found comparable results for tinnitus-related EVs independent of MD with extremely low relative humidity reducing EV risk within one day and a same-day increase in risk under extremely humid conditions. Currently, the scientific literature lacks any leads to how relative humidity could affect tinnitus directly. An infodemiological study reported a winter peak in web-based inquiries for tinnitus [37]. Relative humidity is generally higher in winter as colder air has a decreased capability of holding water vapor and saturates at lower levels than hot air. However, our study did not show seasonal variations in tinnitus-related EVs, which occur mostly due to acute, severe, or debilitating cases of tinnitus, making an effect of relative humidity due to seasons unlikely. Another risk factor for tinnitus is SSNHL. A study on audiogram configurations in patients with SSNHL has suggested a correlation between ascending audiogram patterns and high relative humidity on the day of onset [38]. The authors discuss a potential pathophysiological similarity between MD and SSNHL with ascending patterns due to endolymphatic hydrops based on the overlapping findings on high relative humidity as a risk factor. However, in our study, over 80% of patients with available Weber’s and Rinne’s test assessments did not show indication of hearing loss, albeit not confirmed by pure-tone audiometry due to the emergency setting. As a result, we cannot attribute the association between extreme relative humidity and tinnitus to hearing loss for most of the study population.

In contrast with a reported negative correlation between temperature and tinnitus severity in MD [21], our study found extremely low temperatures to be a mitigating risk factor for tinnitus-related EVs within 5 and 4 days after the single-day events and cold waves, respectively. These findings are further contrasted by a Japanese report of higher rates of MD attacks after the passing of a cold front [39]. Our results, therefore, suggest a differing temperature response in non-MD-related tinnitus cases.

Precipitation and its potential effect on tinnitus has so far not been investigated. Our findings suggest that prolonged extremely high precipitation exerts a mitigating effect on tinnitus-related EVs. Although speculative, the lack of impact of single-day events compared to prolonged precipitation could potentially suggest that the auditory stimulus of prolonged precipitation in the form of rainfall during warmer months has a soothing effect on tinnitus. Sound therapy is one treatment option for patients with chronic tinnitus. Although acoustic stimulation may bring some relief to patients by masking tinnitus symptoms, the treatment approach is not aimed at the underlying cause of tinnitus and high-level evidence is limited. While European guidelines currently do not recommend sound therapy, American guidelines mention sound therapy as an option for patients with bothersome, persistent tinnitus [13, 17]. A recent study employed a smartphone-based noise generator to investigate the benefit of enriching the sound environment of chronic tinnitus patients [40]. Most participants opted to listen to environmental sounds and rainfall. At the 3-month follow-up, participants reported a reduced overall tinnitus severity. Extreme precipitation in the form of prolonged rainfall may, therefore, provide an auditory stimulus leading to transient symptom relief in patients suffering from tinnitus and resulting in a temporary reduction in EV rates.

Lastly, extreme mean wind speeds showed minor late effects on cumulative risk within 12 days, which are unlikely to be clinically relevant. In accordance, wind speed did not correlate with tinnitus severity or attack onset in MD patients [21].

Our study had several limitations. First, the retrospective study design carries inherent bias concerning data availability and homogeneity concerning diagnostic follow-up and whereabouts of patients during extreme weather events. Although EVs of patients who reported acute hearing loss as their primary complaint were excluded, we were unable to distinguish between patients suffering from tinnitus with or without hearing loss due to the lack of pure tone audiometry data. Additionally, seasons and occupations of patients can have a major impact on the degree to which patients are directly exposed to weather conditions. Nevertheless, our study provides new avenues for future studies that are appropriately designed to receive real-time data of patients’ tinnitus symptoms and whereabouts during such events (e.g., smart phone app-based studies). Second, our model considered a lag period of 14 days. While we controlled for demand fluctuations related to weekdays and public holidays, we were unable to control for other potential confounders, such as degree of weather exposure, that may have impacted EV rates during the two weeks after extreme conditions. Next, we could not distinguish between rainfall and snowfall, which is common during winter months in Vienna, based on the available meteorological data. Regarding generalizability, the tinnitus-related EVs took place at a single tertiary care hospital resulting in a study population with acute and more severe cases. Our findings are, therefore, not applicable to all tinnitus cases. Equivalently, this study focused on extreme weather events. Therefore, the reported associations between extreme weather and tinnitus-related EVs are not applicable to moderate weather conditions. Finally, while our study generates potential hypotheses for underlying mechanisms of how extreme meteorological conditions impact EVs for tinnitus, our study was not designed to prove any causative link between the reported associations.

## Conclusion

Extreme weather conditions are associated with changes in tinnitus-related EV rates. Extremely high relative humidity significantly increased same-day RR. Extreme atmospheric pressure and temperature, as well as prolonged extreme precipitation, showed delayed effects on cRR. These findings warrant further clinical and experimental research on how meteorological variables affect tinnitus.

## Supplementary Information

Below is the link to the electronic supplementary material.

Supplementary file1 (DOCX 449 KB)
