# Supplementary material for: Meteorological extremes and their impact on tinnitus-related emergency room visits: a time-series analysis
Source: Eur Arch Otorhinolaryngol. 2023 Mar 1;280(9):3997–4007. doi: 10.1007/s00405-023-07894-1 (PMC9976663; doi:10.1007/s00405-023-07894-1)
Supplement: Supplementary file 1 — Supplementary file1 (DOCX 449 KB) [file 405_2023_7894_MOESM1_ESM.docx]

# Supplementary material

*European Archives of Oto-Rhino-Laryngology*

Meteorological extremes and their impact on tinnitus-related emergency room visits: a time-series analysis

Markus Haas^1^, Mateo Lucic^1^, Franziska Pichler^1^, Alexander Lein^1^, Faris F. Brkic^1^, Dominik Riss^1^ and David T. Liu^1^

^1^ Department of Otorhinolaryngology, Head and Neck Surgery, Medical University of Vienna, Vienna, Austria

**Corresponding author:**

Dominik Riss

Address: Medical University of Vienna, Waehringer Guertel 18-20, 1090 Vienna, Austria.

E-mail: dominik.riss@meduniwien.ac.at

Phone number: +43 1 40400 – 33760


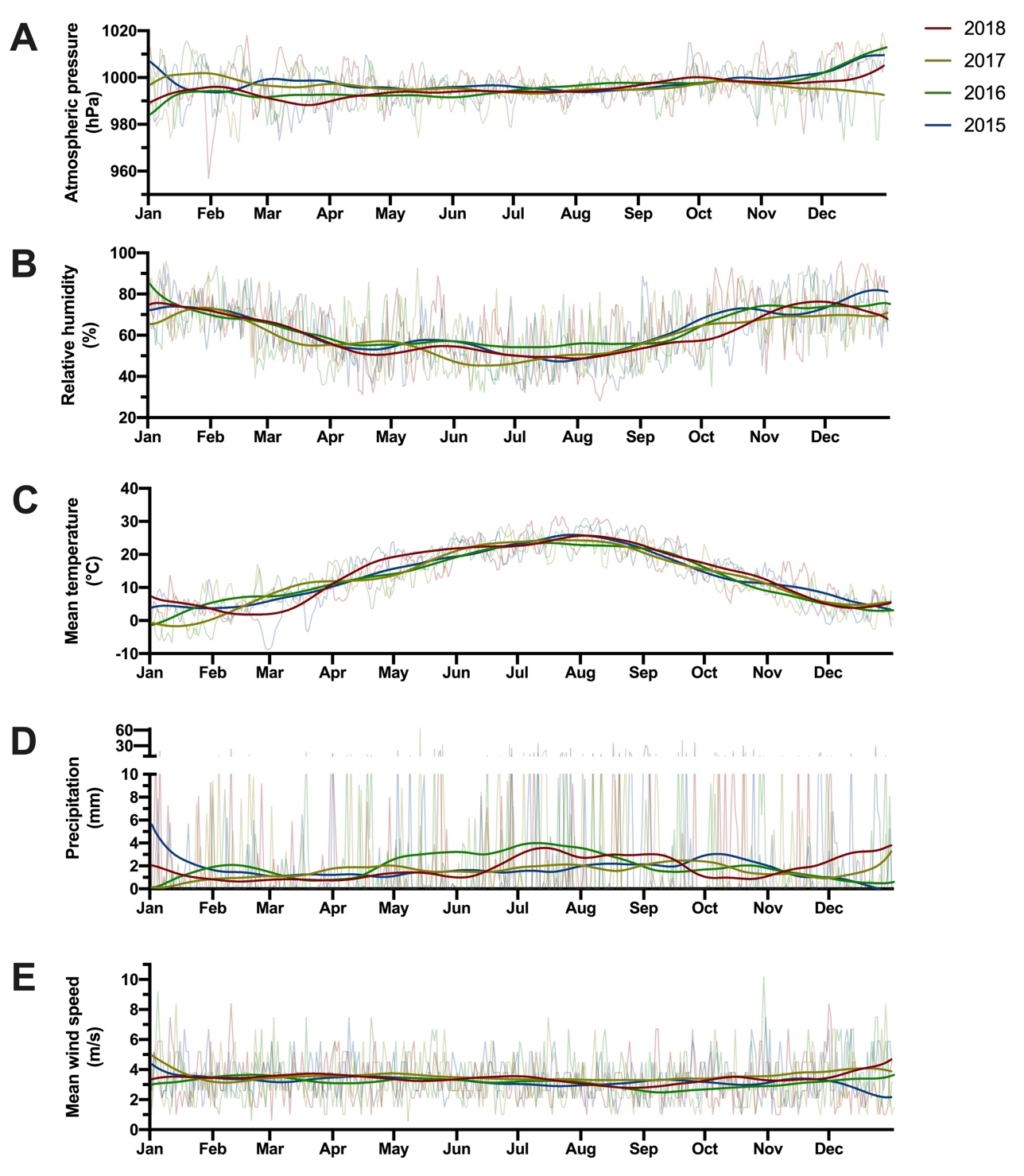


**Supplementary Fig. 1** Atmospheric pressure (A), relative humidity (B), mean temperature (C), precipitation (D) and mean wind speed (E) are shown as LOWESS curves and individual values from 2015 to 2018. The underlying weather data has previously been reported in: Haas M, Lucic M, Pichler F, et al. Extreme weather conditions influence the frequency of epistaxis-related emergency room visits [published online ahead of print, 2022 Nov 9]. Rhinology. 2022. doi:10.4193/Rhin22.342

| **Relative risk (RR)**  after single-day extreme conditions (1 day) | | | | |
| --- | --- | --- | --- | --- |
| **Atmospheric pressure** | **976 hPa (P_1_)** | **983 hPa (P_5_)** | **1009 hPa (P_95_)** | **1014 hPa (P_99_)** |
| lag0 | 1.25 [0.6-2.61; p=0.554] | 1.27 [0.82-1.96; p=0.286] | 0.98 [0.62-1.54; p=0.93] | 1.07 [0.49-2.33; p=0.872] |
| lag1 | 0.71 [0.3-1.67; p=0.434] | 0.73 [0.43-1.25; p=0.252] | 1.25 [0.7-2.24; p=0.446] | 1.42 [0.53-3.83; p=0.49] |
| lag3 | 1.04 [0.62-1.73; p=0.88] | **1.4 [1.04-1.89; p=0.028]** | 1.32 [0.98-1.77; p=0.07] | 1.42 [0.89-2.28; p=0.144] |
| lag7 | 1.12 [0.87-1.44; p=0.368] | 1.06 [0.93-1.21; p=0.386] | 1.03 [0.92-1.15; p=0.604] | 0.88 [0.72-1.08; p=0.226] |
| lag14 | 0.62 [0.34-1.15; p=0.13] | 1.01 [0.8-1.27; p=0.922] | 0.85 [0.7-1.04; p=0.106] | 0.84 [0.62-1.15; p=0.284] |
| **Relative humidity** | **34% (P_1_)** | **39% (P_5_)** | **86% (P_95_)** | **92% (P_99_)** |
| lag0 | 0.52 [0.23-1.15; p=0.106] | 0.99 [0.66-1.49; p=0.98] | 1.4 [0.97-2.02; p=0.068] | **1.75 [1.01-3.03; p=0.046]** |
| lag1 | 0.78 [0.35-1.71; p=0.53] | 0.93 [0.61-1.43; p=0.744] | 0.77 [0.51-1.15; p=0.204] | 0.79 [0.41-1.49; p=0.462] |
| lag3 | 0.79 [0.5-1.24; p=0.306] | 0.82 [0.61-1.11; p=0.2] | 0.78 [0.57-1.05; p=0.102] | 0.67 [0.41-1.1; p=0.116] |
| lag7 | 0.88 [0.71-1.08; p=0.224] | 1.03 [0.9-1.17; p=0.678] | 1.04 [0.92-1.17; p=0.574] | 1.07 [0.88-1.3; p=0.51] |
| lag14 | 1.25 [0.91-1.71; p=0.162] | 0.97 [0.77-1.21; p=0.768] | **0.79 [0.63-0.99; p=0.038]** | 0.94 [0.68-1.3; p=0.696] |
| **Mean temperature** | **-5°C (P_1_)** | **0°C (P_5_)** | **27°C (P_95_)** | **30°C (P_99_)** |
| lag0 | 1.17 [0.23-5.89; p=0.85] | 1.02 [0.43-2.45; p=0.958] | 0.72 [0.3-1.76; p=0.47] | 0.64 [0.2-2.03; p=0.444] |
| lag1 | 0.76 [0.07-7.95; p=0.82] | 0.52 [0.16-1.67; p=0.272] | 3.01 [0.88-10.32; p=0.08] | 3.54 [0.7-18.04; p=0.128] |
| lag3 | 0.67 [0.23-2.01; p=0.48] | 0.69 [0.37-1.26; p=0.224] | 1.48 [0.84-2.61; p=0.17] | 1.86 [0.86-4.04; p=0.116] |
| lag7 | 1.08 [0.8-1.46; p=0.62] | 1 [0.83-1.2; p=0.974] | 0.88 [0.74-1.06; p=0.168] | 0.84 [0.66-1.06; p=0.132] |
| lag14 | 0.84 [0.5-1.4; p=0.496] | 0.95 [0.68-1.34; p=0.788] | 1.01 [0.73-1.39; p=0.962] | 1.01 [0.67-1.53; p=0.956] |
| **Precipitation** | - | - | **10mm (P_95_)** | **24mm (P_99_)** |
| lag0 | - | - | 0.82 [0.57-1.18; p=0.276] | 1 [0.44-2.23; p=0.994] |
| lag1 | - | - | 1.15 [0.82-1.63; p=0.42] | 0.36 [0.11-1.12; p=0.078] |
| lag3 | - | - | 0.83 [0.62-1.11; p=0.202] | 0.81 [0.4-1.63; p=0.556] |
| lag7 | - | - | 1.06 [0.92-1.22; p=0.396] | 0.99 [0.66-1.49; p=0.958] |
| lag14 | - | - | 1.04 [0.82-1.32; p=0.756] | 1.02 [0.53-1.94; p=0.958] |
| **Mean wind speed** | **1m/s (P_1_)** | **2m/s (P_5_)** | **6m/s (P_95_)** | **8m/s (P_99_)** |
| lag0 | 1.48 [0.99-2.2; p=0.056] | 1.09 [0.96-1.23; p=0.202] | 1 [0.68-1.48; p=0.984] | 0.71 [0.21-2.47; p=0.592] |
| lag1 | 0.74 [0.48-1.13; p=0.166] | 0.94 [0.82-1.08; p=0.358] | 1.26 [0.87-1.82; p=0.23] | 0.12 [0-4.77; p=0.256] |
| lag3 | 0.84 [0.58-1.21; p=0.346] | 1.03 [0.92-1.15; p=0.622] | **1.36 [1.01-1.84; p=0.046]** | 0.65 [0.28-1.53; p=0.322] |
| lag7 | 0.92 [0.74-1.13; p=0.416] | 0.95 [0.9-1.01; p=0.122] | 0.9 [0.76-1.08; p=0.272] | **1.52 [1.02-2.25; p=0.038]** |
| lag14 | 1.02 [0.74-1.39; p=0.91] | 0.95 [0.86-1.04; p=0.258] | 0.99 [0.75-1.32; p=0.95] | 0.67 [0.3-1.52; p=0.342] |

**Supplementary Table 1.** Relative risk for tinnitus-related EV under extreme weather conditions (1^st^, 5^th^, 95^th^, and 99^th^ percentile) compared to median conditions of atmospheric pressure, relative humidity, mean temperature, precipitation and mean wind speed. Confidence intervals (95%) and p-values are shown in brackets. Significant results (p≤0.05) are highlighted in bold print and grey.

| **Cumulative relative risk (cRR)**  after single-day extreme conditions (1 day) | | | | |
| --- | --- | --- | --- | --- |
| **Atmospheric pressure** | **976 hPa (P_1_)** | **983 hPa (P_5_)** | **1009 hPa (P_95_)** | **1014 hPa (P_99_)** |
| lag0 | 1.25 [0.6-2.61; p=0.554] | 1.27 [0.82-1.96; p=0.286] | 0.98 [0.62-1.54; p=0.93] | 1.07 [0.49-2.33; p=0.872] |
| lag0-1 | 0.89 [0.39-2.03; p=0.778] | 0.93 [0.57-1.52; p=0.772] | 1.23 [0.77-1.96; p=0.388] | 1.51 [0.73-3.12; p=0.26] |
| lag0-3 | 1.01 [0.35-2.9; p=0.982] | 1.51 [0.87-2.6; p=0.14] | 1.4 [0.89-2.22; p=0.148] | 1.31 [0.6-2.83; p=0.5] |
| lag0-7 | 2.58 [0.57-11.68; p=0.218] | **2.37 [1.12-5.03; p=0.024]** | 1.38 [0.74-2.6; p=0.31] | 0.65 [0.2-2.14; p=0.48] |
| lag0-14 | 0.31 [0.02-4.23; p=0.384] | **3.24 [1.04-10.15; p=0.044]** | 1.9 [0.75-4.83; p=0.178] | 0.86 [0.14-5.23; p=0.87] |
| **Relative humidity** | **34% (P_1_)** | **39% (P_5_)** | **86% (P_95_)** | **92% (P_99_)** |
| lag0 | 0.52 [0.23-1.15; p=0.106] | 0.99 [0.66-1.49; p=0.98] | 1.4 [0.97-2.02; p=0.068] | **1.75 [1.01-3.03; p=0.046]** |
| lag0-1 | **0.4 [0.16-1; p=0.05]** | 0.93 [0.58-1.49; p=0.752] | 1.08 [0.69-1.69; p=0.742] | 1.37 [0.71-2.65; p=0.344] |
| lag0-3 | **0.28 [0.11-0.77; p=0.014]** | 0.67 [0.37-1.2; p=0.176] | 1.09 [0.64-1.86; p=0.74] | 1.34 [0.63-2.83; p=0.448] |
| lag0-7 | **0.27 [0.08-0.85; p=0.026]** | 0.71 [0.33-1.52; p=0.38] | 0.92 [0.46-1.88; p=0.828] | 0.92 [0.28-3.02; p=0.896] |
| lag0-14 | **0.12 [0.03-0.56; p=0.006]** | 0.54 [0.18-1.63; p=0.274] | 0.48 [0.17-1.35; p=0.162] | 1.22 [0.26-5.83; p=0.802] |
| **Mean temperature** | **-5°C (P_1_)** | **0°C (P_5_)** | **27°C (P_95_)** | **30°C (P_99_)** |
| lag0 | 1.17 [0.23-5.89; p=0.85] | 1.02 [0.43-2.45; p=0.958] | 0.72 [0.3-1.76; p=0.47] | 0.64 [0.2-2.03; p=0.444] |
| lag0-1 | 0.89 [0.19-4.18; p=0.884] | 0.53 [0.22-1.27; p=0.154] | 2.17 [0.9-5.21; p=0.082] | 2.25 [0.7-7.19; p=0.172] |
| lag0-3 | 0.58 [0.16-2.1; p=0.406] | 0.87 [0.43-1.74; p=0.686] | 1.06 [0.51-2.19; p=0.876] | 1.17 [0.46-2.93; p=0.744] |
| lag0-7 | 0.35 [0.09-1.37; p=0.132] | **0.41 [0.18-0.97; p=0.042]** | 1.24 [0.52-2.95; p=0.632] | 1.12 [0.36-3.5; p=0.85] |
| lag0-14 | 0.52 [0.1-2.78; p=0.442] | 0.53 [0.17-1.68; p=0.282] | 0.79 [0.24-2.67; p=0.708] | 0.99 [0.18-5.33; p=0.99] |
| **Precipitation** | - | - | **10mm (P_95_)** | **24mm (P_99_)** |
| lag0 | - | - | 0.82 [0.57-1.18; p=0.276] | 1 [0.44-2.23; p=0.994] |
| lag0-1 | - | - | 1.15 [0.82-1.63; p=0.42] | 0.36 [0.11-1.12; p=0.078] |
| lag0-3 | - | - | 0.83 [0.62-1.11; p=0.202] | 0.81 [0.4-1.63; p=0.556] |
| lag0-7 | - | - | 1.06 [0.92-1.22; p=0.396] | 0.99 [0.66-1.49; p=0.958] |
| lag0-14 | - | - | 1.04 [0.82-1.32; p=0.756] | 1.02 [0.53-1.94; p=0.958] |
| **Mean wind speed** | **1m/s (P_1_)** | **2m/s (P_5_)** | **6m/s (P_95_)** | **8m/s (P_99_)** |
| lag0 | 1.48 [0.99-2.2; p=0.056] | 1.09 [0.96-1.23; p=0.202] | 1 [0.68-1.48; p=0.984] | 0.71 [0.21-2.47; p=0.592] |
| lag0-1 | 1.09 [0.61-1.94; p=0.768] | 1.02 [0.85-1.22; p=0.836] | 1.26 [0.75-2.11; p=0.378] | 0.08 [0-4.06; p=0.21] |
| lag0-3 | 1.03 [0.45-2.34; p=0.944] | 1.11 [0.87-1.42; p=0.404] | 1.37 [0.67-2.79; p=0.386] | 0.03 [0-2.03; p=0.104] |
| lag0-7 | 0.85 [0.24-2.96; p=0.798] | 0.97 [0.66-1.43; p=0.874] | 1 [0.34-2.92; p=0.998] | 0.09 [0-8.06; p=0.292] |
| lag0-14 | 0.39 [0.06-2.65; p=0.336] | 0.67 [0.37-1.22; p=0.192] | 1.22 [0.24-6.25; p=0.808] | 0.11 [0-27.04; p=0.434] |

**Supplementary Table 2.** Cumulative relative risk for tinnitus-related EV under extreme weather conditions (1^st^, 5^th^, 95^th^, and 99^th^ percentile) compared to median conditions of atmospheric pressure, relative humidity, mean temperature, precipitation and mean wind speed. Confidence intervals (95%) and p-values are shown in brackets. Significant results (p≤0.05) are highlighted in bold print and grey.

| **Relative risk (RR)**  after prolonged extreme conditions (3 days) | | | | |
| --- | --- | --- | --- | --- |
| **Atmospheric pressure**  over 3 days (mean) | **980 hPa (P_1_)** | **985 hPa (P_5_)** | **1008 hPa (P_95_)** | **1013 hPa (P_99_)** |
| lag0 | 1.6 [0.87-2.96; p=0.13] | 1.4 [0.93-2.12; p=0.106] | 1.09 [0.68-1.75; p=0.706] | 1.69 [0.76-3.78; p=0.2] |
| lag1 | 0.49 [0.17-1.39; p=0.18] | 0.59 [0.29-1.2; p=0.146] | 0.91 [0.39-2.1; p=0.824] | 0.62 [0.15-2.51; p=0.506] |
| lag3 | 1.25 [0.89-1.75; p=0.194] | 1.24 [0.99-1.55; p=0.066] | 1.11 [0.86-1.44; p=0.412] | 1.14 [0.75-1.73; p=0.544] |
| lag7 | 1.08 [0.97-1.2; p=0.176] | 1.07 [1-1.14; p=0.064] | 1.07 [0.99-1.15; p=0.076] | 0.95 [0.84-1.08; p=0.416] |
| lag14 | **0.79 [0.63-0.99; p=0.038]** | 1 [0.89-1.13; p=0.998] | 0.92 [0.82-1.03; p=0.136] | 0.89 [0.74-1.07; p=0.208] |
| **Relative humidity**  over 3 days (mean) | **37% (P_1_)** | **41% (P_5_)** | **82% (P_95_)** | **89% (P_99_)** |
| lag0 | 0.73 [0.43-1.26; p=0.26] | 0.98 [0.68-1.4; p=0.904] | 1.2 [0.83-1.72; p=0.33] | 1.4 [0.83-2.36; p=0.212] |
| lag1 | 0.78 [0.36-1.68; p=0.532] | 0.9 [0.53-1.53; p=0.694] | 0.86 [0.51-1.48; p=0.594] | 0.78 [0.35-1.72; p=0.532] |
| lag3 | 0.78 [0.57-1.09; p=0.146] | 0.81 [0.64-1.01; p=0.06] | 0.82 [0.65-1.03; p=0.086] | 0.72 [0.5-1.02; p=0.068] |
| lag7 | 0.94 [0.84-1.05; p=0.252] | 1.03 [0.96-1.11; p=0.438] | 1.05 [0.98-1.13; p=0.162] | 1.02 [0.92-1.14; p=0.674] |
| lag14 | 1.13 [0.96-1.32; p=0.136] | 0.98 [0.87-1.11; p=0.774] | **0.88 [0.78-1; p=0.042]** | 0.94 [0.79-1.12; p=0.504] |
| **Mean temperature**  over 3 days (mean) | **-4°C (P_1_)** | **0°C (P_5_)** | **26°C (P_95_)** | **30°C (P_99_)** |
| lag0 | 1.15 [0.2-6.57; p=0.874] | 1.15 [0.39-3.38; p=0.802] | 0.55 [0.18-1.65; p=0.284] | 0.68 [0.15-3.14; p=0.62] |
| lag1 | 0.34 [0.01-9.98; p=0.532] | 0.43 [0.05-3.43; p=0.428] | 4.34 [0.57-33.03; p=0.156] | 3.53 [0.2-63.76; p=0.394] |
| lag3 | 0.52 [0.19-1.37; p=0.186] | 0.7 [0.39-1.28; p=0.25] | **1.83 [1.02-3.3; p=0.044]** | **2.61 [1.15-5.89; p=0.022]** |
| lag7 | 1.1 [0.89-1.36; p=0.376] | 0.99 [0.86-1.15; p=0.928] | **0.85 [0.74-0.98; p=0.026]** | **0.78 [0.64-0.95; p=0.014]** |
| lag14 | 0.9 [0.67-1.2; p=0.47] | 0.98 [0.79-1.2; p=0.824] | 0.92 [0.75-1.15; p=0.474] | 1.01 [0.76-1.34; p=0.948] |
| **Precipitation**  over 3 days (sum) | - | - | **24mm (P_95_)** | **40mm (P_99_)** |
| lag0 | - | - | 0.85 [0.61-1.19; p=0.344] | 1.22 [0.71-2.09; p=0.47] |
| lag1 | - | - | **0.62 [0.41-0.94; p=0.026]** | 1.08 [0.56-2.08; p=0.81] |
| lag3 | - | - | 0.88 [0.71-1.09; p=0.232] | 0.96 [0.65-1.4; p=0.816] |
| lag7 | - | - | 1 [0.92-1.09; p=0.982] | 1.16 [1-1.34; p=0.052] |
| lag14 | - | - | 0.99 [0.85-1.15; p=0.856] | 0.87 [0.65-1.16; p=0.334] |
| **Mean wind speed**  over 3 days (mean) | **1m/s (P_1_)** | **2m/s (P_5_)** | **5m/s (P_95_)** | **6m/s (P_99_)** |
| lag0 | 1.35 [0.75-2.41; p=0.316] | 1.05 [0.89-1.24; p=0.566] | 1.11 [0.86-1.45; p=0.414] | 1.02 [0.62-1.66; p=0.946] |
| lag1 | 0.83 [0.42-1.63; p=0.582] | 0.92 [0.75-1.14; p=0.456] | 0.93 [0.66-1.33; p=0.71] | 0.71 [0.38-1.33; p=0.288] |
| lag3 | 0.97 [0.64-1.47; p=0.884] | 0.98 [0.87-1.09; p=0.69] | 1.04 [0.88-1.24; p=0.616] | 1.29 [0.97-1.71; p=0.082] |
| lag7 | 1.07 [0.9-1.27; p=0.462] | 0.97 [0.93-1.01; p=0.18] | 0.98 [0.91-1.05; p=0.566] | 1.01 [0.88-1.16; p=0.916] |
| lag14 | 0.89 [0.64-1.24; p=0.49] | **0.88 [0.81-0.95; p<0.001]** | **0.86 [0.77-0.97; p=0.01]** | 1.03 [0.82-1.29; p=0.792] |

**Supplementary Table 3.** Relative risk for tinnitus-related EV under extreme weather conditions over three days (1^st^, 5^th^, 95^th^, and 99^th^ percentile) compared to median conditions of atmospheric pressure, relative humidity, mean temperature, precipitation and mean wind speed. The percentiles for extreme conditions were calculated using the three-day average for atmospheric pressure, relative humidity, mean temperature and mean wind speed or the three-day sum for precipitation. Confidence intervals (95%) and p-values are shown in brackets. Significant results (p≤0.05) are highlighted in bold print and grey.

| **Cumulative relative risk (cRR)**  after prolonged extreme conditions (3 days) | | | | |
| --- | --- | --- | --- | --- |
| **Atmospheric pressure**  over 3 days (mean) | **980 hPa (P_1_)** | **985 hPa (P_5_)** | **1008 hPa (P_95_)** | **1013 hPa (P_99_)** |
| lag0 | 1.6 [0.87-2.96; p=0.13] | 1.4 [0.93-2.12; p=0.106] | 1.09 [0.68-1.75; p=0.706] | 1.69 [0.76-3.78; p=0.2] |
| lag0-1 | 0.79 [0.42-1.48; p=0.458] | 0.83 [0.54-1.27; p=0.388] | 1 [0.61-1.63; p=0.986] | 1.05 [0.47-2.34; p=0.9] |
| lag0-3 | 1.42 [0.93-2.17; p=0.1] | **1.42 [1.09-1.85; p=0.01]** | **1.36 [1.04-1.77; p=0.026]** | 1.45 [0.92-2.3; p=0.112] |
| lag0-7 | **2.38 [1.34-4.23; p=0.002]** | **2.1 [1.49-2.96; p<0.001]** | **1.47 [1.06-2.04; p=0.022]** | 0.87 [0.46-1.64; p=0.666] |
| lag0-14 | 1.19 [0.47-3; p=0.71] | **2.54 [1.51-4.25; p<0.001]** | **2.04 [1.26-3.3; p=0.004]** | 1.1 [0.42-2.86; p=0.848] |
| **Relative humidity**  over 3 days (mean) | **37% (P_1_)** | **41% (P_5_)** | **82% (P_95_)** | **89% (P_99_)** |
| lag0 | 0.73 [0.43-1.26; p=0.26] | 0.98 [0.68-1.4; p=0.904] | 1.2 [0.83-1.72; p=0.33] | 1.4 [0.83-2.36; p=0.212] |
| lag0-1 | 0.57 [0.32-1.02; p=0.056] | 0.88 [0.6-1.28; p=0.498] | 1.03 [0.72-1.48; p=0.854] | 1.08 [0.64-1.84; p=0.77] |
| lag0-3 | **0.35 [0.22-0.55; p<0.001]** | **0.59 [0.43-0.79; p<0.001]** | 1.03 [0.78-1.35; p=0.856] | 1.27 [0.87-1.85; p=0.214] |
| lag0-7 | **0.37 [0.22-0.63; p<0.001]** | 0.71 [0.48-1.03; p=0.072] | 1.02 [0.72-1.45; p=0.91] | 0.93 [0.53-1.61; p=0.788] |
| lag0-14 | **0.19 [0.09-0.38; p<0.001]** | **0.45 [0.26-0.77; p=0.004]** | 0.72 [0.45-1.17; p=0.186] | 0.81 [0.39-1.66; p=0.562] |
| **Mean temperature**  over 3 days (mean) | **-4°C (P_1_)** | **0°C (P_5_)** | **26°C (P_95_)** | **30°C (P_99_)** |
| lag0 | 1.15 [0.2-6.57; p=0.874] | 1.15 [0.39-3.38; p=0.802] | 0.55 [0.18-1.65; p=0.284] | 0.68 [0.15-3.14; p=0.62] |
| lag0-1 | 0.39 [0.06-2.8; p=0.352] | 0.5 [0.15-1.68; p=0.26] | 2.38 [0.74-7.59; p=0.144] | 2.39 [0.46-12.55; p=0.302] |
| lag0-3 | 0.76 [0.37-1.57; p=0.46] | 0.93 [0.59-1.47; p=0.758] | 0.87 [0.53-1.43; p=0.592] | 0.94 [0.49-1.82; p=0.864] |
| lag0-7 | **0.37 [0.19-0.74; p=0.004]** | **0.47 [0.29-0.78; p=0.004]** | 1.01 [0.6-1.68; p=0.982] | 0.97 [0.48-1.96; p=0.938] |
| lag0-14 | 0.51 [0.23-1.16; p=0.11] | 0.57 [0.3-1.08; p=0.086] | 0.6 [0.3-1.18; p=0.14] | 0.85 [0.32-2.28; p=0.748] |
| **Precipitation**  over 3 days (sum) | - | - | **24mm (P_95_)** | **40mm (P_99_)** |
| lag0 | - | - | 0.85 [0.61-1.19; p=0.344] | 1.22 [0.71-2.09; p=0.47] |
| lag0-1 | - | - | **0.53 [0.36-0.78; p=0.002]** | 1.32 [0.74-2.36; p=0.346] |
| lag0-3 | - | - | **0.43 [0.28-0.65; p<0.001]** | 1.25 [0.63-2.47; p=0.522] |
| lag0-7 | - | - | **0.49 [0.28-0.85; p=0.012]** | 1.21 [0.45-3.25; p=0.71] |
| lag0-14 | - | - | **0.3 [0.13-0.7; p=0.006]** | 3.04 [0.66-14; p=0.154] |
| **Mean wind speed**  over 3 days (mean) | **1m/s (P_1_)** | **2m/s (P_5_)** | **5m/s (P_95_)** | **6m/s (P_99_)** |
| lag0 | 1.35 [0.75-2.41; p=0.316] | 1.05 [0.89-1.24; p=0.566] | 1.11 [0.86-1.45; p=0.414] | 1.02 [0.62-1.66; p=0.946] |
| lag0-1 | 1.11 [0.6-2.06; p=0.738] | 0.97 [0.82-1.15; p=0.718] | 1.04 [0.79-1.38; p=0.77] | 0.73 [0.41-1.3; p=0.278] |
| lag0-3 | 1.52 [0.76-3.06; p=0.238] | 1.07 [0.89-1.28; p=0.476] | 1.06 [0.78-1.43; p=0.726] | 0.77 [0.4-1.49; p=0.43] |
| lag0-7 | 2.45 [0.89-6.75; p=0.084] | 0.96 [0.73-1.26; p=0.772] | 0.91 [0.57-1.44; p=0.676] | 0.74 [0.3-1.81; p=0.508] |
| lag0-14 | 1.12 [0.22-5.7; p=0.89] | **0.53 [0.34-0.81; p=0.004]** | 0.65 [0.32-1.31; p=0.228] | 1.55 [0.42-5.7; p=0.512] |

**Supplementary Table 4.** Cumulative relative risk for tinnitus-related EV under extreme weather conditions over three days (1^st^, 5^th^, 95^th^, and 99^th^ percentile) compared to median conditions of atmospheric pressure, relative humidity, mean temperature, precipitation and mean wind speed. The percentiles for extreme conditions were calculated using the three-day average for atmospheric pressure, relative humidity, mean temperature and mean wind speed or the three-day sum for precipitation. Confidence intervals (95%) and p-values are shown in brackets. Significant results (p≤0.05) are highlighted in bold print and grey.
